# A chromosome-level genome assembly of *Solanum chilense*, a tomato wild relative associated with resistance to salinity and drought

**DOI:** 10.3389/fpls.2024.1342739

**Published:** 2024-03-08

**Authors:** Corentin Molitor, Tomasz J. Kurowski, Pedro M. Fidalgo de Almeida, Zoltan Kevei, Daniel J. Spindlow, Steffimol R. Chacko Kaitholil, Justice U. Iheanyichi, H. C. Prasanna, Andrew J. Thompson, Fady R. Mohareb

**Affiliations:** ^1^ The Bioinformatics Group, School of Water, Energy and Environment, Cranfield University, Wharley End, United Kingdom; ^2^ Soil, Agrifood and Biosciences, Cranfield University, Wharley End, United Kingdom; ^3^ Division of Vegetable Crops, ICAR-Indian Institute of Horticultural Research, Bangalore, India

**Keywords:** Genome assembly, *S. chilense*, BUSCO, drought, salt, transcriptome

## Abstract

**Introduction:**

*Solanum chilense* is a wild relative of tomato reported to exhibit resistance to biotic and abiotic stresses. There is potential to improve tomato cultivars via breeding with wild relatives, a process greatly accelerated by suitable genomic and genetic resources.

**Methods:**

In this study we generated a high-quality, chromosome-level, *de novo* assembly for the *S. chilense* accession LA1972 using a hybrid assembly strategy with ~180 Gbp of Illumina short reads and ~50 Gbp long PacBio reads. Further scaffolding was performed using Bionano optical maps and 10x Chromium reads.

**Results:**

The resulting sequences were arranged into 12 pseudomolecules using Hi-C sequencing. This resulted in a 901 Mbp assembly, with a completeness of 95%, as determined by Benchmarking with Universal Single-Copy Orthologs (BUSCO). Sequencing of RNA from multiple tissues resulting in ~219 Gbp of reads was used to annotate the genome assembly with an RNA-Seq guided gene prediction, and for a *de novo* transcriptome assembly. This chromosome-level, high-quality reference genome for *S. chilense* accession LA1972 will support future breeding efforts for more sustainable tomato production.

**Discussion:**

Gene sequences related to drought and salt resistance were compared between *S. chilense* and *S. lycopersicum* to identify amino acid variations with high potential for functional impact. These variants were subsequently analysed in 84 resequenced tomato lines across 12 different related species to explore the variant distributions. We identified a set of 7 putative impactful amino acid variants some of which may also impact on fruit development for example the *ethylene-responsive transcription factor WIN1* and *ethylene-insensitive protein 2*. These variants could be tested for their ability to confer functional phenotypes to cultivars that have lost these variants.

## Highlights

- This article describes the first chromosome-level genome assembly for the tomato wild-type relative *Solanum chilense.*
- A hybrid assembly strategy was followed to generate 12 pseudomolecule assembly with 95% completeness levels
- Genes related to drought and salt resistance were studied, and resulted in the identification of seven putative impactful amino acid variants, some of which have an impact on fruit development.

## Introduction

Non-starchy vegetables are one of the cornerstones of a healthy human diet (Newman, 2021). Domesticated tomato (*Solanum lycopersicum* L.) is the most-consumed non-starchy vegetable in the world, reaching 180 million tonnes of production in 2019, equivalent to every human eating 63 g of tomato every day of the year (FAO, 2021). Tomato breeding to maintain and enhance yield, resilience, sustainability, and nutrition of tomato crops is therefore an important endeavour. There has been concern that modern breeding leads to a reduction in genetic diversity, leading to less resilience against shifting pest and disease risks and lower human nutrition in favour of yield. At least in The Netherlands, this was a temporary problem that started to reverse in the 1970s, with an eightfold increase in genetic diversity that delivered greater disease resistance and improved fruit quality via the introduction of DNA from wild species into cultivated tomato (Schouten et al., 2019).

The tomato reference genome (Tomato Genome, 2012) and short-read resequencing (100 Tomato Genome Sequencing Consortium et al., 2014) provide a wealth of SNP and InDel polymorphism data across most wild species, whereas long-read platforms have been used to improve assemblies (Zhou and Pichersky, 2020) and report variants (Gao et al., 2019) and structural variants (Alonge et al., 2020) in tomato and its most closely related sub-species and wild species, *S. lycopersicum* var. *cerasiforme*, *S. pimpinellifolium*, *S. cheesmaniae*, and *S. galapagense*. Long reads have also been used to create a “graph pan genome” (Zhou et al., 2022), but only for cultivated tomato and its most closely related wild species (*S. lycopersicum* var. *cerasiforme* and *S. pimpinellifolium*). A chromosome-level assembly is also reported for the close relative *S. pimpinellifolium* (Wang et al., 2020). For more distantly related species, there are a few high-quality, chromosome-level assemblies available: *S. pennellii* (Bolger et al., 2014), *S. sitiens* (Molitor et al., 2021), and *S. lycopersicoides* (Powell et al., 2022). These provide the genomic resources to facilitate gene functional studies and marker discoveries needed for wide introgression breeding.

**Figure 1:**
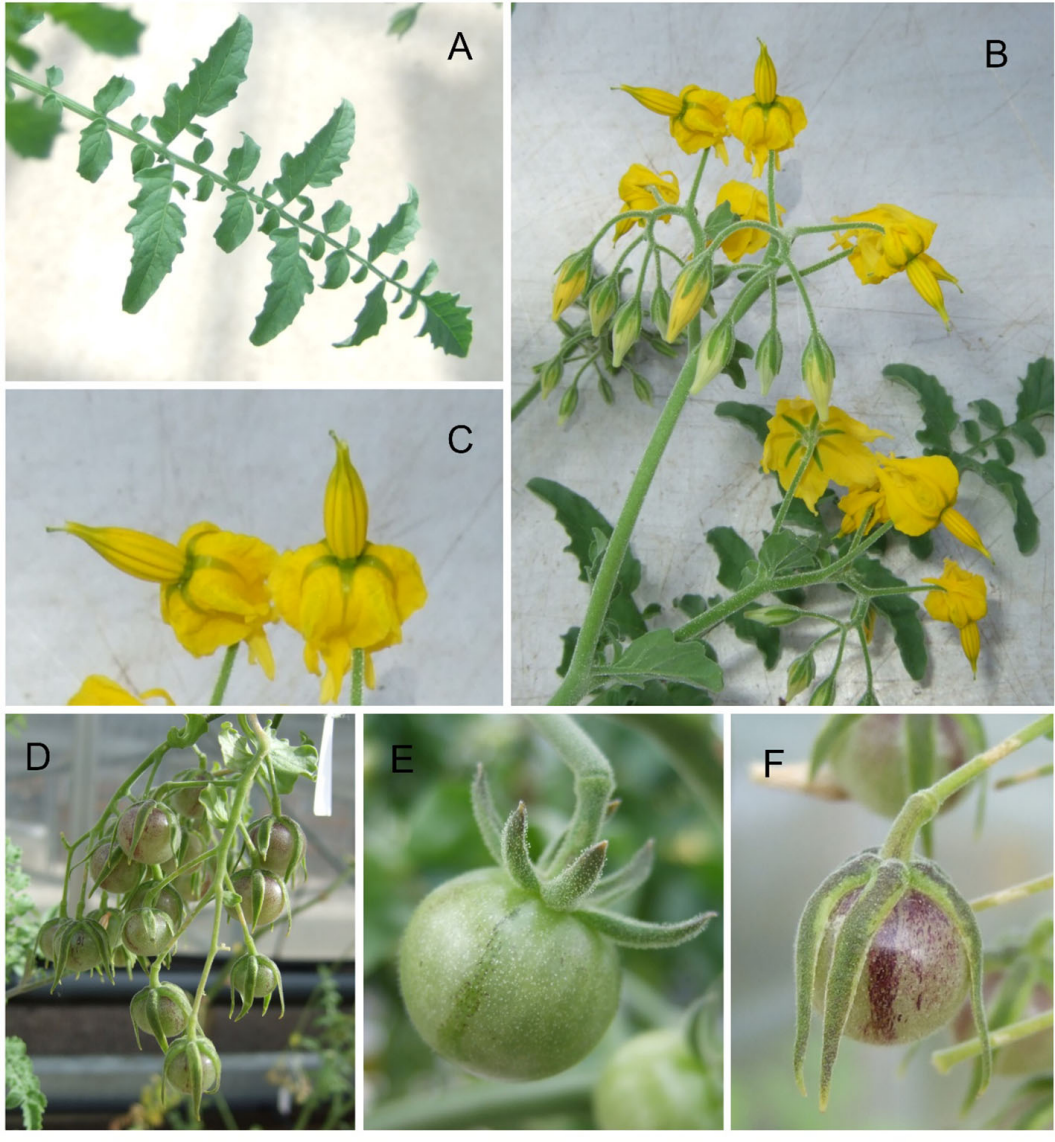
Images of plants of Solanum chilense LA1972 growing in a glasshouse. **(A)** Fully expanded leaf; **(B)** inflorescence; **(C)** detail of flowers from image **(B, D)** truss of ripening fruit; **(E)** single fruit at green ripe stage; **(F)** single ripe fruit. Twenty plants were mass-sibling pollinated to achieve the fruit set.

*S. chilense* is a wild relative of tomato, classified into the *Solanum* section *Lycopersicon* in the Eriopersicon group along with *S. habrochaites*, *S. huaylasense*, *S. corneliomulleri*, and *S. peruvianum* (Peralta et al., 2008); population genetic studies estimate that it diverged from *S. peruvianum* less than 0.55 million years ago (Stadler et al., 2008). It is diploid (*n* = 12) allogamous and self-incompatible; successful crossing with cultivated tomato is very rare (Rick, 1979), requiring bridging lines or embryo rescue. It is native to southern Peru and northern Chile where it can grow in altitudes ranging from sea level to over 3,500 m (Chetelat et al., 2008; Moyle, 2008) and is often found “in the extremely dry high-elevation deserts of the western Andean slope … and in the unique lomas habitat” where lomas are “small areas of vegetation occurring as islands in a sea of hyper-arid desert” (Peralta et al., 2008). The species characteristically has greyish pubescent leaves, straight anther tubes with exerted stigma, long erect peduncles, and inedible green fruits of ~1 cm coated in short trichomes when immature and developing a purple stripe when mature (**Figure 1**). *S. chilense* is considered, based on its ecological distribution, to be resistant to extreme environments, including drought, high salinity, and low-temperature stresses (Moyle, 2008; Nakazato et al., 2010). A physiological study claimed salinity resistance for *S. chilense* LA4107 and its F1 hybrid with cultivated tomato (Bigot et al., 2023); another study described the higher salt stress tolerance of LA4107 over *S. lycopersicum* by analysing the water status and antioxidant enzymes under high NaCl stress (Martínez et al., 2020), while the increased ethylene production of LA4107 during stress was promoting the salt adaptation via maintained stomatal conductance (Gharbi et al., 2017). Moreover, *S. chilense* is resistant to pathogens, notably the *Tomato Yellow Leaf Curl Virus*, the *Cucumber Mosaic Virus* (Chetelat et al., 2009), and the *Tomato Mottle Virus* (Ji et al., 2007). Moreover, *S. chilense* also has great ability to mitigate pathogen infections; notably, the LA1969 line possess the resistant *Ty-1*/*Ty-3* alleles against the *Tomato Yellow Leaf Curl Virus* (Verlaan et al., 2013), and this resistance is also influenced by the *SLMAPK3* expression that regulates the salicylic acid and jasmonic acid signalling pathways (Li et al., 2017). The transgenic *S. chilense* allele of the *pcht28* chitinase gene generated improved resistance to *Verticillium dahliae*, a common fungal disease in tomato (Tabaeizadeh et al., 1999). The *S. chilense Cucumber Mosaic Virus* resistance locus was introgressed and mapped to the tomato chromosome 12 (Stamova and Chetelat, 2000), while *Tomato Mottle Virus* resistance was linked to the *Ty-6* on chromosome 10, a major resistance locus of *S. chilense* LA2779 against begomoviruses (Gill et al., 2019). Three common tomato pathogen species—*Alternaria solani*, *Phytophthora infestans*, and *Fusarium oxysporium*—were also tested on different *S. chilense* populations, and the results showed large variations and mosaic resistance patterns that were unrelated to their geographic locations (Stam et al., 2017), where the quantitative variation of *Phytophthora* resistance between and within the natural *S. chilense* populations is predominantly determined by the plant genotype (Kahlon et al., 2021).

Although leaves or whole plants of *S. pennellii* are more resistant to desiccation compared to cultivated tomato, the same was not considered to be true for *S. chilense*, where it was hypothesised that its drought resistance is due to the foraging ability of its root system in rocky riverbeds, rather than leaf traits (Rick, 1973). It was also shown that *S. chilense* adapts better to the arid condition during the plant development by effective regulation of morphological and physiological traits when compared to tomato cultivars (Tapia et al., 2016). These changes were further studied in six different S. *chilense* accessions, and the physiological analyses coupled with the gene expression data also revealed that the drought and heat resistance of these lines is less related to their natural environment and phylogenetic relations, while it is more associated with their particular growth habit (Blanchard-Gros et al., 2021).

Böndel et al. (2015) demonstrated that in the southern range, *S. chilense* shows high genetic variation between the coastal and high-altitude populations, suggesting different local adaptation between the eastern and western sides of the Atacama desert (Böndel et al., 2015). The high level of heterozygosity arising from the allogamous, self-incompatible mating system creates a challenge to generate a high-quality reference genome. A scaffold-level assembly for *S. chilense* (accession LA3111) is the only genome assembly reported so far for this species; the data allowed the identification of two unique coiled-coil domain containing NLR subfamilies: CNL20 and CNL21 (Stam et al., 2019).

The aim of this work was to produce a high-quality reference genome for *S. chilense* LA1972 using a hybrid-sequencing strategy including both Illumina short reads and Pacific Biosciences long reads, followed by Bionano optical mapping and Hi-C sequencing to orient and order the scaffolds into pseudomolecules. Additionally, the generated genome assembly has been complemented with a high-quality and functionally annotated *de novo* transcriptome assembly with gene models. Our assembly provides a resource that will underpin the introgression of beneficial traits into cultivated tomato.

We chose *S. chilense* LA1972 because it was collected from an extremely dry environment and is classified as “drought tolerant” in the C.M. Rick, Tomato Genetics Resource Centre (TGRC) catalogue, and because of the availability of successful embryo-rescued progeny from crossing with cultivated tomato for the development of genetic resources.

## Materials and methods

### Plant materials

*S. chilense* LA1972 seeds were obtained from the UC Davis/C.M. Rick TGRC maintained by the Department of Plant Sciences, University of California, Davis, CA 95616, USA. LA3111 from which the scaffold level assembly was reported (Stam et al., 2019) was collected from Tarata, Tacna province at elevation 3,070 m; LA1972 was collected from Rio Sama, also Tacna province at elevation 650 m; these two locations are approximately 70 km apart.

Twenty plants were raised and crossed between siblings to bulk seeds for physiological analysis, but one individual plant was used for all DNA and RNA extractions—this plant is maintained through clonal propagation at Cranfield University, UK. Seed from *S. lycopersicum* cv. Kashi Amrit was obtained from the Division of Crop Improvement, ICAR-Indian Institute of Vegetable Research, Varanasi, India.

### DNA and RNA extraction

*S. chilense* leaves used for DNA and RNA extraction were obtained from plant grown to flowering stage in a glasshouse facility at Cranfield University, UK. The DNeasy and RNeasy Plant Mini Kits (Qiagen, Manchester, UK) were used to prepare genomic DNA and total RNA for Illumina sequencing according to the manufacturer’s instructions. High-molecular-weight (HMW) genomic DNA was extracted for PacBio sequencing and Bionano optical mapping. The HMW DNA was prepared by the Earlham Institute, Norwich, UK using the Bionano Prep™ Plant Tissue DNA isolation kit.

### Sequencing data

Two PCR-free, paired-end Illumina libraries were prepared and sequenced on an Illumina HiSeq2500™ platform at the Earlham Institute (UK), with a read length of 250 base pairs (bp) and a mean insert size of 395 bp. The whole genome sequencing yielded a total of ~180 Gbp and the quality of the reads was assessed with FASTQC v0.11.

Pacific Bioscience long reads were obtained from two different platforms, namely, RS-II and Sequel. Using RS-II, ~16 Gbp of data were generated in 2,186,914 reads, with an N50 of 9,384 bp. The longest read was 49,532 bp long. For the Sequel platform, ~34 Gbp of data were generated in 3,818,160 reads, with an N50 of 14,770 bp. The longest read was 160,787 bp long. For each platform, the bam files were converted into a multi-sequence fasta file, containing all the reads. The two resulting fasta files were concatenated into one, which was used in the subsequent assembly steps.

Optical maps were generated with the BioNano Irys platform, at the Earlham Institute, yielding ~314 Gbp of molecules larger than 100 kbp. The *Bss*SI restriction enzyme was used and resulted in a label density of ~11 per 100 kbp.

A single library of Paired-End 10× Chromium, generating 28 Gbp of data, was sequenced at the Earlham Institute following 10× Genomics guidelines for genomes between 0.1 and 1.6 Gbp. The fastq files were processed with the “basic” pipeline from LongRanger v2.2.2, which interleaved the two fastq files and performed quality control (read trimming, barcode error correction, and barcode whitelisting). The 10× molecule barcode, present in the first 16 bp of each left read, was removed and added to the corresponding read pair identifiers, which is required for most downstream analyses. Finally, the Arcs pipeline (Yeo et al., 2018), used as part of the assembly process, requires the 10× reads to have the barcode as part of their name: this was achieved with a custom Perl script “10x_custom_script.pl” (see the Data availability statement). Reads without a barcode were removed, which corresponded to ~17 million reads, representing 5% of the total number.

Finally, chromosome conformation Hi-C data were generated using the Arima-HiC kit, according to the manufacturer’s protocol (Arima Genomics, San Diego, US). A Hi-C library was prepared using the Arima approach, which uses a mixture of restriction enzymes cutting chromatin at the following sequence motifs: ^GATC, G^ANTC, G^TNA, and T^TAA. The library was sequenced on an Illumina HiSeq X™ platform, which yielded a total of 367,560,717 read pairs (2 × 150 bp). The quality of the library preparation was assessed by Arima Genomics using human control GM cells, which identified ~56% of long-range *cis* interactions, ~24% of *trans* interactions, and 0.2% duplication.

For gene model prediction and functional annotation, 15 Illumina 125-bp paired-end RNA-Seq libraries were sequenced (HiSeq2500™) generating 219 Gbp of data. The 15 tissue samples were: 2 × fruit and sepals at 14 days after pollination (dap); fruit at 36 dap; sepals at 36 dap; fruit at 46; root with or without dehydration treatment; fully expanded leaf with or without dehydration treatment; 2 ×flower; 2 × stem; senescing leaf; young expanding leaf and meristem combined. Deyhdration treatments were achieved by drying tissue on the laboratory bench under ambient conditions until 10% of fresh weight was lost (inducing loss of turgor except in stem).

The quality of the RNA-Seq reads was assessed with FastQC. The correction of erroneous K-mers was performed using RCorrector v1.0.3.1 (Song and Florea, 2015), a tool that utilises a K-mer spectrum-based method to convert rare K-mers (a K-mer size of 19 was used) into those that are more commonly found within the assembly. Reads deemed unfixable by RCorrector were removed using FilterUncorrectablePEfastq.py from the TranscriptomeAssemblyTools package. Bases with a PHRED score < 5 were trimmed and adapter sequences and trimmed reads below a length of 100 bases were removed using Trim Galore (Bioinformatics B), a wrapper around Cutadapt (Martin, 2011).

### Genome size estimation

**Figure 2:**
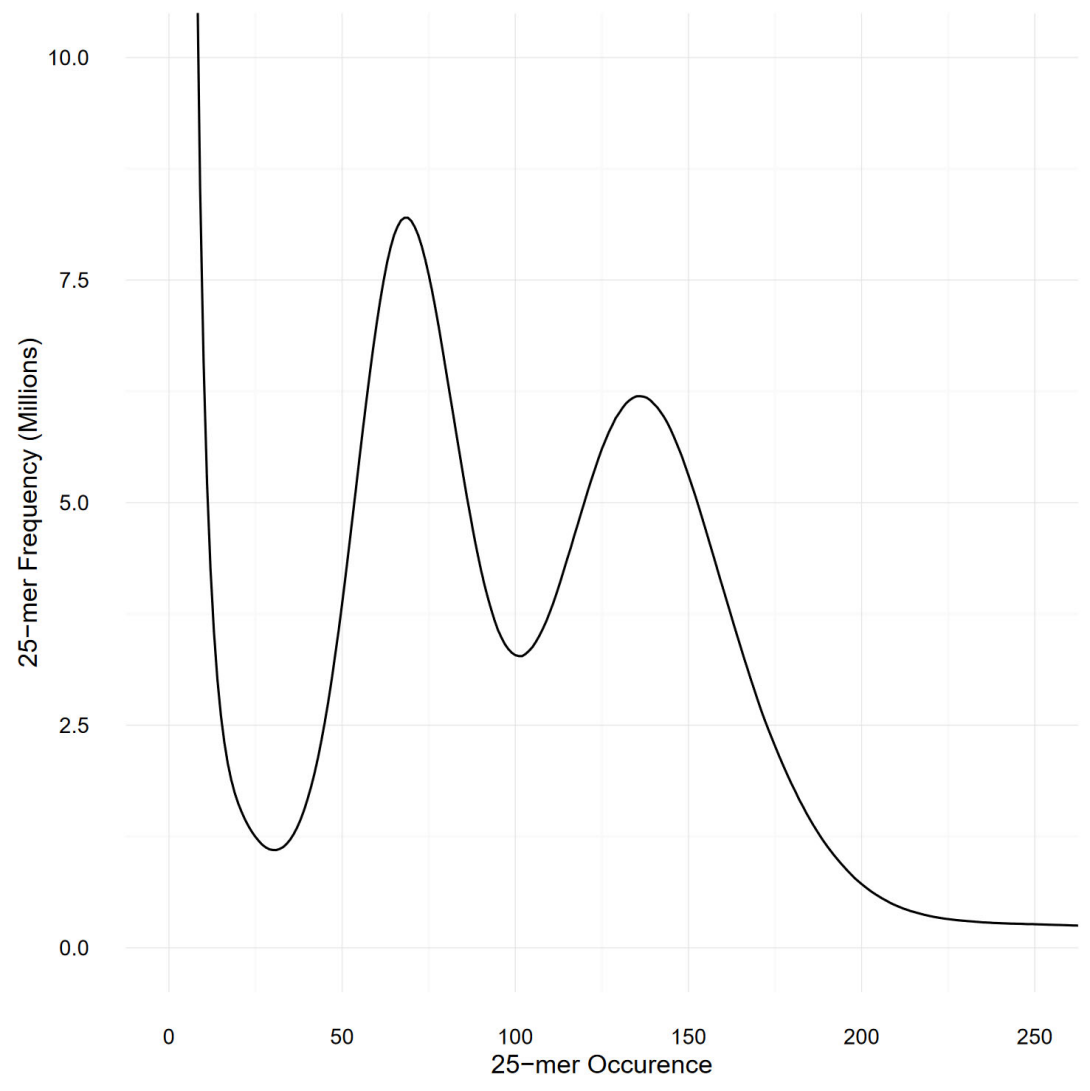
Histogram representing the frequency of the 25-mers present in both Illumina libraries, as counted by Jellyfish. The two peaks, at occurrence 68 and 136, respectively represents the heterozygous and homozygous peaks and demonstrate the high heterozygosity present in our *S. chilense* sample.

The size of the genome was estimated by performing a k-mer-based analysis using the Illumina short reads (Williams et al., 2013): 25-mers from the two Illumina libraries were counted with Jellyfish v2.2.3 (Marçais and Kingsford, 2011) with the -C parameter to consider both strands. A total of 133 billion 25-mers were counted and plotted as a histogram (**Figure 2**). The 18 billion 25-mers with an occurrence lower than 30 were considered artifacts (as they probably spawned from sequencing errors) and were disregarded during the genome size estimation.

### *De novo* assembly strategy

The hybrid assembler MaSuRCA v3.2.7 (Zimin et al., 2013) generated the *de novo* contig assembly, based on both the paired-end Illumina reads and the combined RS-II and Sequel PacBio reads. The k-mer size of 127 was automatically determined by MaSuRCA, the ploidy was set to 2 and the k-mer count threshold was set to 2 as the Illumina coverage was more than 100×. The Jellyfish hash size was set to 125 billion and 64 threads were used to speed up computation time; the default values were kept for the remaining parameters.

Despite the satisfactory contiguity, the number of artifact duplications was high, as assessed by BUSCO (Manni et al., 2021) and resulted in larger-than-expected total genome size (see the Genome size estimation section). This is expected when attempting to assemble a heterozygous, out-breeding, wild species as it is the case here. Redundans v0.13a (Pryszcz and Gabaldón, 2016) was applied to the contig assembly with the Illumina reads, in order to remove artifact duplications and then scaffold the resulting reduced assembly. Next, SSPACE v1-1 (Boetzer et al., 2011) further scaffolded the assembly, using the default parameters and the combined PacBio long reads (RSII and Sequel).

Optical maps from BioNano Genomics, obtained with the BssSI (GACGAG) restriction enzyme served as input to the “Hybrid Scaffold” pipeline, which super-scaffolded the assembly. The conflict filter levels (options *-B* and *-N*) were set to 1 and the xml file describing the remaining parameters (option *-c*) is available at https://github.com/MCorentin/Solanum_chilense_assembly. The restriction enzyme used in this analysis was manually added to Hybrid Scaffold, as it is not supported by default. This conservative tool removes all unmapped scaffolds from the assembly, which reduces the completeness, hence the Perl script “hybridScaffold_finish_fasta.pl” (Shelton et al., 2015) was run to reintegrate the discarded scaffolds into the assembly.

The super-scaffolded assembly was given as input to Arcs v8.25 (Yeo et al., 2018) and LINKS v1.8.6 pipeline (Warren et al., 2015), which uses long-range information from the 10× Chromium reads in order to further scaffold the assembly. First, the interleaved 10× reads were aligned to the super-scaffolded assembly with bwa v0.7.17 (Li and Durbin, 2009) using the “mem” algorithm. Then, a Graphviz Dot file, representing scaffolds as nodes and evidence that two scaffolds are linked as edges, was generated with Arcs. The following parameters were chosen: the minimum sequence identity for read alignment was set to 95% (option -s), the range for the barcode multiplicity was set to 30–10,000 (option -m), and default values were kept for the remaining parameters. The *makeTSVfile.py* python script translated the Graphviz Dot file to a tsv file, which contains all possible oriented sequence pairs with the number of supporting barcodes. This tsv file was given as input to LINKS, with default parameters except for the k-mer size, which was set to 20, to generate the super-scaffolded fasta file.

Assembly polishing was performed via two iterations of Pilon v1.22 (Walker et al., 2014). For each iteration, first the Illumina short reads were aligned to the super-scaffolded assembly with *bwa mem*, then the resulting SAM file was converted to a BAM file, sorted, and indexed with SAMtools v1.9 (Li et al., 2009). Pilon was run on the assembly fasta file with the aligned BAM files and the following parameters: *–changes*, to generate a log file listing all the changes, and *–fix all*, to fix individual base errors, small InDels (insertion/deletion), gap sizes, and local misassemblies.

The polished assembly was inputted to BBmap’s *dedupe.sh* script v37.72 to remove duplicated sequences from the assembly, based on sequence similarity. For this step, *storequality* was set to false; *absorbrc* was set to true to absorb reverse complements as well as normal orientation; *touppercase* was set to true to avoid mismatches due to lowercases; *minidentity*, representing the minimum sequence similarity to consider two sequences as duplicated, was set to 90%; *minlengthpercent* and *minoverlappercent* were both set to 0 to ignore filtering based on contig lengths and overlap; the maximum number of allowed substitutions, *maxsubs*, and InDels, *maxedits*, were set to 40,000 and 1,000, respectively; and finally, the seed length, *k*, was set to 31. The values for *maxsubs* and *maxedits* were chosen empirically after testing a range of different values and assessing the resulting assembly with Quast and BUSCO (See **Supplementary Table 9**).

The final step of the scaffolding was done with GapFiller v1-10 (Nadalin et al., 2012), which harnessed information from the paired-end Illumina reads to resize and fill gaps between or within scaffolds. The minimum number of overlapping bases with the edge of the gap was set to 30 (option -m), and default values were kept for the remaining parameters, notably the number of iterations, which was set to 10.

**Figure 3:**
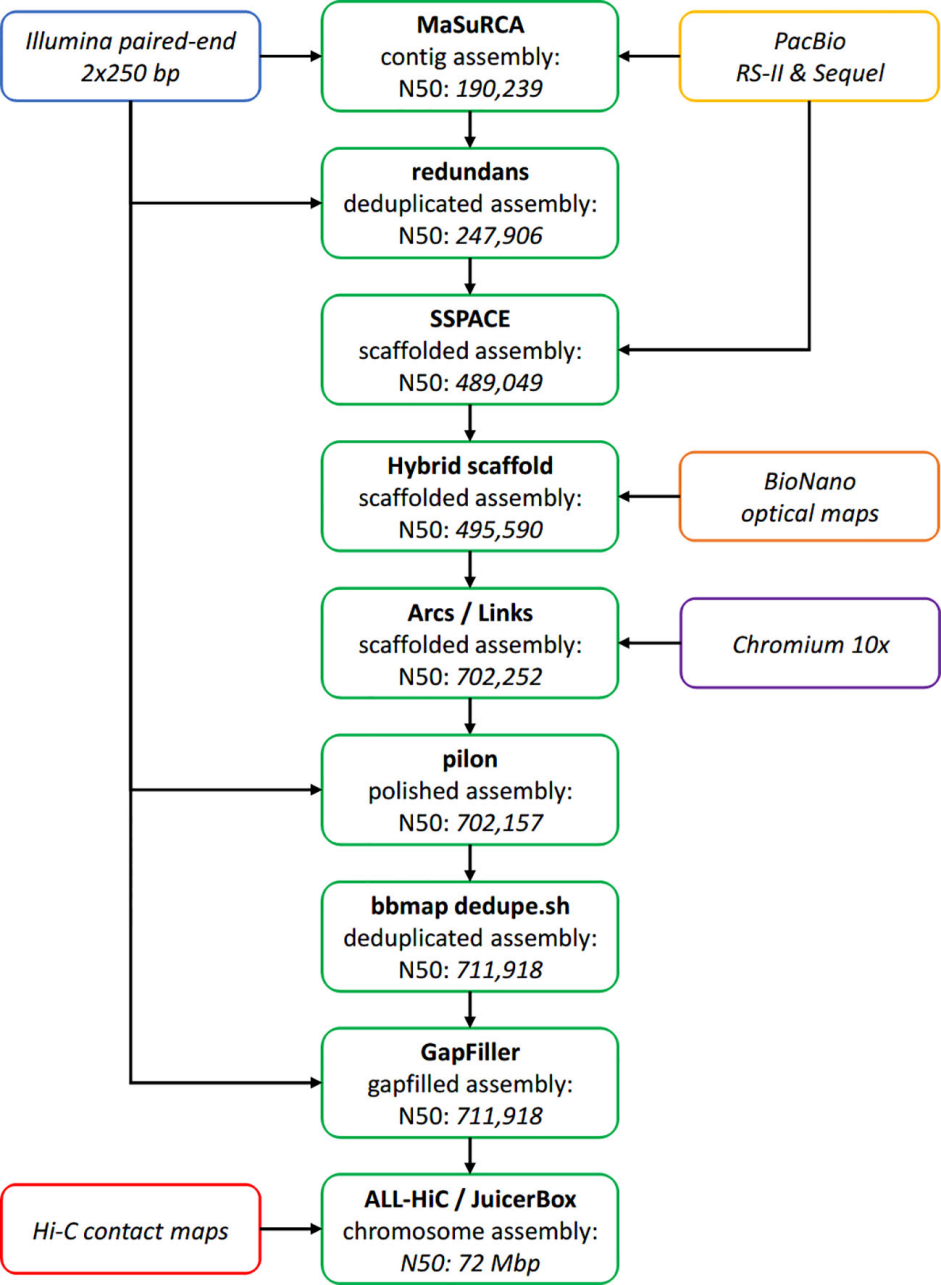
Overview of the assembly pipeline. Assembly steps are represented in green, with the resulting N50 specified inside the box (in base pairs, unless stated otherwise).

An overview of the whole assembly pipeline is available as **Figure 3**.

### Construction of pseudomolecules from the Hi-C data

Contact information, obtained from mapping the Hi-C reads against the assembly, was used to orient and order the scaffolds into chromosome-sized sequences. First, the Hi-C reads were trimmed with Trimmomatic v0.39 (Bolger et al., 2014) using a sliding window of 4 bases, and trimming the reads when the average base quality reached below 20 (SLIDINGWINDOW:4:20), as well as removing reads smaller than 50 bases (MINLEN:50). The trimmed reads were mapped to the gapfilled assembly with bwa v0.7.17 using the “*-SP*” option to align the pairs as independent single-end reads, while keeping all the appropriate pair-related flags in the resulting SAM file, and the “*-5*” option to only keep alignments with the smallest coordinates as primary, when dealing with split alignments. This last option is beneficial when aligning Hi-C data, as it reduces the number of secondary mappings and the “noise” from the Hi-C alignment. The resulting SAM file was filtered with SAMtools v1.9 (Li et al., 2009) to remove reads in non-primary, supplementary, and unpaired alignments. The SAM file was further processed with the *PreprocessSAMs.pl* script from LACHESIS (Burton et al., 2013) to remove redundant, chimeric, and uninformative read pairs, while retaining significant Hi-C links from the alignment. This step reduces the file size and, subsequently, the I/O time needed to process it.

The ALL-HiC pipeline v0.9.13 (Zhang et al., 2019) oriented and ordered the scaffolds in the assembly. First, the Hi-C reads were aligned against the corrected assembly, using the same parameters (*-SP* and *-5*) and post-processing the SAM file with the same options as before, but with an additional step of removing the alignments with a quality lower than 40 (*samtools view -q 40*). Then, the *ALLHiC_partition* script clustered the scaffolds into 12 groups (*-k 12*) as the number of expected chromosomes in the *S. chilense* genome. After the partition step, the *extract* step created ChromLinkMatrix files containing the intrachromosomal links data for each cluster. These ChromLinkMatrix files were given as input to the *optimize* step to find the orientation and ordering best supported by the Hi-C alignments.

We identified some misjoins in our pseudomolecules, based on comparisons against chromosomes from closely related species, namely, *S. lycopersicum* (Hosmani et al., 2019) and *S. pennellii* (Bolger et al., 2014). Juicebox v1.11.08 (Durand et al., 2016) was used to manually curate the orientation and order of the scaffolds in these regions. First, the *agp* file obtained from ALL-HiC was converted to an *assembly* format with the *agp2assembly.py* script from *phasegenomics*, then a *hic* file was created using *matlock*, from the alignment of the Hi-C reads against the corrected genome. Finally, the reviewed chromosome-level assembly was converted back into a fasta file with the *juicebox_assembly_converter.py* script.

### Quality assessment

An important step in the generation of a *de novo* reference genome is the quality assessment of the final assembly. Assembly quality metrics were calculated with Quast v4.5 (Gurevich et al., 2013). Completeness and duplication levels were measured with BUSCO v5.3.2 (Manni et al., 2021) against the OrthoDB *Solanaceae* v10 (Kriventseva et al., 2019), containing 3,052 highly conserved orthologues from this family. Sequence similarity with closely related species, *S. lycopersicum v4.0* and *S. pennellii* was assessed with Mummer v4.0.0 (Marçais et al., 2018) using the *–mum* and *-c 1000* parameters, to remove noise from the global alignment. Finally, the K-mer Analysis Toolkit (KAT) v2.4.0 (Mapleson et al., 2017) assessed the completeness of the assembly by comparing 27-mers obtained from the assembly against those obtained from the Illumina reads.

### Gene prediction and annotation

Genes were predicted from the final assembly using Augustus v3.3 (Stanke and Morgenstern, 2005) with hints obtained via the alignment of the RNA-Seq reads against the assembly. First, repeats present in the final assembly were masked with RepeatMasker version open-4.0.9 (Smit et al., 2013) using the *repeats_master.fasta* library of repeats for *S. lycopersicum* [obtained from SolGenomics (Fernandez-Pozo et al., 2015)]. The *–xsmall* parameter was used to return repetitive regions in lowercases, rather than mask them, which would hinder gene prediction.

Then, the RNA-Seq reads were aligned to the masked assembly with STAR v2.6.0c (Dobin et al., 2013). Both libraries were aligned with default parameters. The two resulting BAM files were merged with SAMtools and then sorted by query with *samtools sort -n*. The *filterBam* script from Augustus was applied to the sorted BAM file with the *–uniq* and *–paired* parameters to remove the background noise from the alignment. Finally, the hints file, containing information about introns, was generated with the *bam2hints* script from Augustus, using the aforementioned BAM file as input.

Gene prediction was performed with Augustus, which was run with the following parameters: *tomato* was chosen as the species; *softMasking* was set to *on*, to indicate that the assembly was soft-masked; *allow_hinted_splicesites* was set to *atac*, to allow Augustus to predict the rare introns that start with AT and end with AC; and *–alternatives-from-evidence* was set to true to allow the prediction of alternative splicing. The default configuration file for the extrinsic evidence, which lists the used sources for the hints and their “boni” and “mali”, was replaced with *–extrinsicCfgFile.*

The genes predicted with Augustus were annotated using OmicsBox v1.3.11 (Conesa and Götz, 2008). The amino acid sequences were blasted against the NCBI-nr database using the *blastp* algorithm, using the following parameters: the *expectation p-value* was set to 1.0e^−3^, the *word size* was set to 5, the *HSP length cutoff* was set to 15, and the *low complexity filter* was turned on. Gene Ontology (GO) mapping and annotation were also performed by OmicsBox based on the blast results. An InterProScan (Jones et al., 2014) search against all available databases was performed on the FASTA sequences with OmicsBox via the web service offered by the EBI.

### Organelles assemblies and annotations

The assembly of *S. chilense* chloroplast and mitochondrial genomes is described in the **Supplementary Materials**.

### *De novo* transcriptome assembly

A *de novo* transcriptome assembly was generated from the RNA-Seq reads using Trinity v2.8.5 (Grabherr et al., 2011) with a k-mer size of 25. *In silico* normalisation was performed by setting the maximum reads coverage to 50× to speed up the process. After completion of the transcriptome assembly, the redundancy was reduced by clustering similar transcripts with CD-HIT-EST v4.8.1 (Li and Godzik, 2006) using a word size of 10 and a sequence identity threshold of 0.95. To remove sequencing artefacts presenting as lowly expressed transcripts, abundance estimation was performed using Trinity’s *align_and_estimate_abundance.pl* and *abundance_estimates_to_matrix.pl* scripts; ultimately, a threshold of 1 TPM was selected, and corresponding transcripts were filtered with Trinity’s *filter_low_expr_transcripts.pl* script.

As for the main assembly, completeness of the transcriptome was assessed at each stage throughout the comparison of orthologues within the assembly to the *Solanaceae* orthoDB dataset using BUSCO. Additionally, completeness was further assessed by realigning the RNA-Seq reads back to the assembly using Bowtie2 v2.3.3.1 (Langmead and Salzberg, 2012). Indeed, assembled transcripts may not fully represent the RNA-Seq reads from which they are derived from and thus alignments from properly and improperly paired reads were captured to quantify read representation.

### Transcriptome functional annotation

The final transcriptome assembly was blasted using *blastx-fast* (Altschul et al., 1990) with default parameters against NCBI’s non-redundant proteins database (NR) and manually generated databases from *S. lycopersicum* (*ITAG3.2*), *S. pennellii* (*Spenn-v2-aa-annot.fa*) annotated proteins, obtained via the SolGenomics website, and The Arabidopsis Information Resources’ annotated protein list (*TAIR10*) (Berardini et al., 2015).

The resultant top 20 Blast hits were loaded into OmicsBox v1.3.11, with an HSP cutoff of 33. GO mapping was performed, after which annotation was run with a cutoff length of 55, a GO weighting of 5, and an e-value filter of 1×10^-6^. Enzyme code mapping was performed for the identification of enzyme codes based on the GO IDs. Finally, an InterProScan search was done on the assembly to detect GO terms based on protein signatures.

### Comparative genomics

A comparative genomics analysis was performed on 84 accessions across 12 tomato species focusing on genes related to drought and salt response. The rationale behind this analysis was to identify variants between *S. lycopersicum* and *S. chilense*, and further wild relatives, which could help us to understand potential differences in drought and salt stress resistance, with the hypothesis that some of these traits had been lost during domestication of *S. lycopersicum*.

First, genes related to drought and salt response were selected, using a set of 16 GO terms, listed in **Table 1**. The terms were obtained from searching the keywords *salt, salinity, water*, and *drought* in the annotated gene list of our *S. chilense* assembly (see the Gene prediction and annotation section).

**Table 1:** List of Gene Ontology terms related to water and salt response, from the S. chilense annotation, obtained by keyword search.

GO ID	GO term
GO:0006833	Water transport
GO:0009414	Response to water deprivation
GO:0009415	Response to water
GO:0009651	Response to salt stress
GO:0009819	Drought recovery
GO:0015250	Water channel activity
GO:0042538	Hyperosmotic salinity response
GO:0042631	Cellular response to water deprivation
GO:0050891	Multicellular organismal water homeostasis
GO:0071472	Cellular response to salt stress
GO:0080148	Negative regulation of response to water deprivation
GO:1901000	Regulation of response to salt stress
GO:1901001	Negative regulation of response to salt stress
GO:1901002	Positive regulation of response to salt stress
GO:1902584	Positive regulation of response to water deprivation
GO:2000070	Regulation of response to water deprivation

The sequence of the annotated genes with the aforementioned GO terms in *S. chilense* was extracted with SeqKit v2.3.0 (Shen et al., 2016). Orthologues in *S. lycopersicum* were identified via a blast search using blast+ v2.13.0, with the blastn algorithm and the *qcov_hsp_perc* parameter set to 90. Multiple sequence alignments of the protein sequences were performed with MAFFT v7.490 (Katoh et al., 2019). An in-house Python script was used to extract the amino acid substitutions, insertions, and deletions from the multiple sequence alignment files.

Finally, PROVEAN (PROtein Variant Effect ANalyzer) v1.1.5 (Choi et al., 2012), SIFT4G (Vaser et al., 2016), and PPVED (Gou et al., 2022) predicted whether an amino acid substitution affected protein function. The PROVEAN analysis was based on blast+ v2.4.0, the nr database v2.4, and CD-HIT v4.6.1 (Li and Godzik, 2006). The SIFT4G analysis was used with UNIPROT’s uniref90 as the database.

## Results and discussion

### Genome size estimation

**Figure 2** represents the k-mer spectra, plotted as a histogram. The number of remaining 25-mers was divided by the expected homozygous coverage, of 136, as determined by the location of the homozygous peak, and revealed an estimated genome size of 845 Mbp. However, the high heterozygous sequence of the sample might have impacted the accuracy of this result. Detailed statistics about the genome size estimation can be found in **Supplementary Table 7**.

### The genome assembly

**Figure 4:**
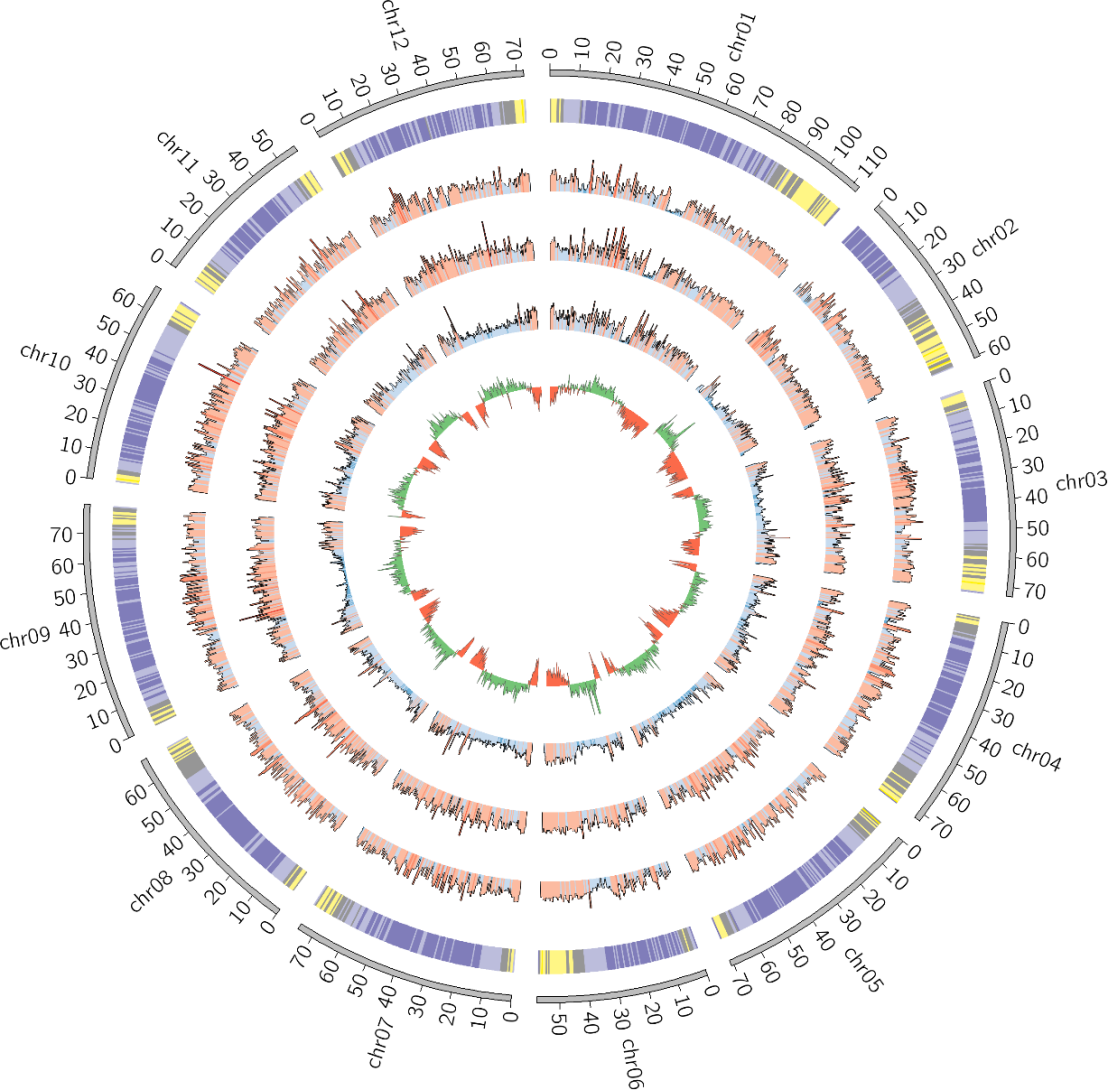
Circos plot of the final S. chilense assembly (scaffolds unmapped to pseudomolecules were omitted). From the outside to the inside, the layers are respectively representing chromosomes, gene density (purple = low density, yellow = high density), SNP density against S. lycopersicum, SNP density against S. pennellii, SNP density against S. chilense LA3111 (blue = low, red = high), and GC content (green = GC content above the genome mean GC content, red = GC content below the genome mean).

The assembly statistics, as measured by Quast, were computed at each step of the pipeline and the results are available in **Table 2**. Detailed statistics are available in **Supplementary Table 1**. The final assembly was also represented as a Circos plot (**Figure 4**).

**Table 2:** Statistics of the assembly at each step of the pipeline, obtained with Quast v4.5.

Stage	Length (Mbp)	# contigs /scaffolds	Largest scaffold (bp)	N50 (bp)
MaSuRCA (contigs)	1,001	9,780	2,713,768	190,239
Redundans	900	6,346	2,971,814	247,906
SSPACE	913	3,066	3,721,406	489,049
Hybrid Scaffold	914	3,057	3,721,406	495,590
Arcs + LINKS	914	2,379	3,811,166	702,252
Pilon	914	2,379	3,808,466	702,157
Bbmap dedupe	901	1,987	3,808,466	711,918
GapFiller	901	1,987	3,809,012	711,918
HiC corrected assembly	901	9,461	2,833,999	212,100
ALL-HiC + Juicer (chromosomes)	902	12 + 1,906	112,267,598	72,333,043
**Final assembly**	**901**	**12 + 1,899**	**112,267,598**	**72,333,043**

The final assembly corresponds to the assembly after removing scaffolds corresponding to organelles and those reported as “to exclude” from the SRA report.The bold values corresponds to the statistics of the final assembly.

**Figure 5:**
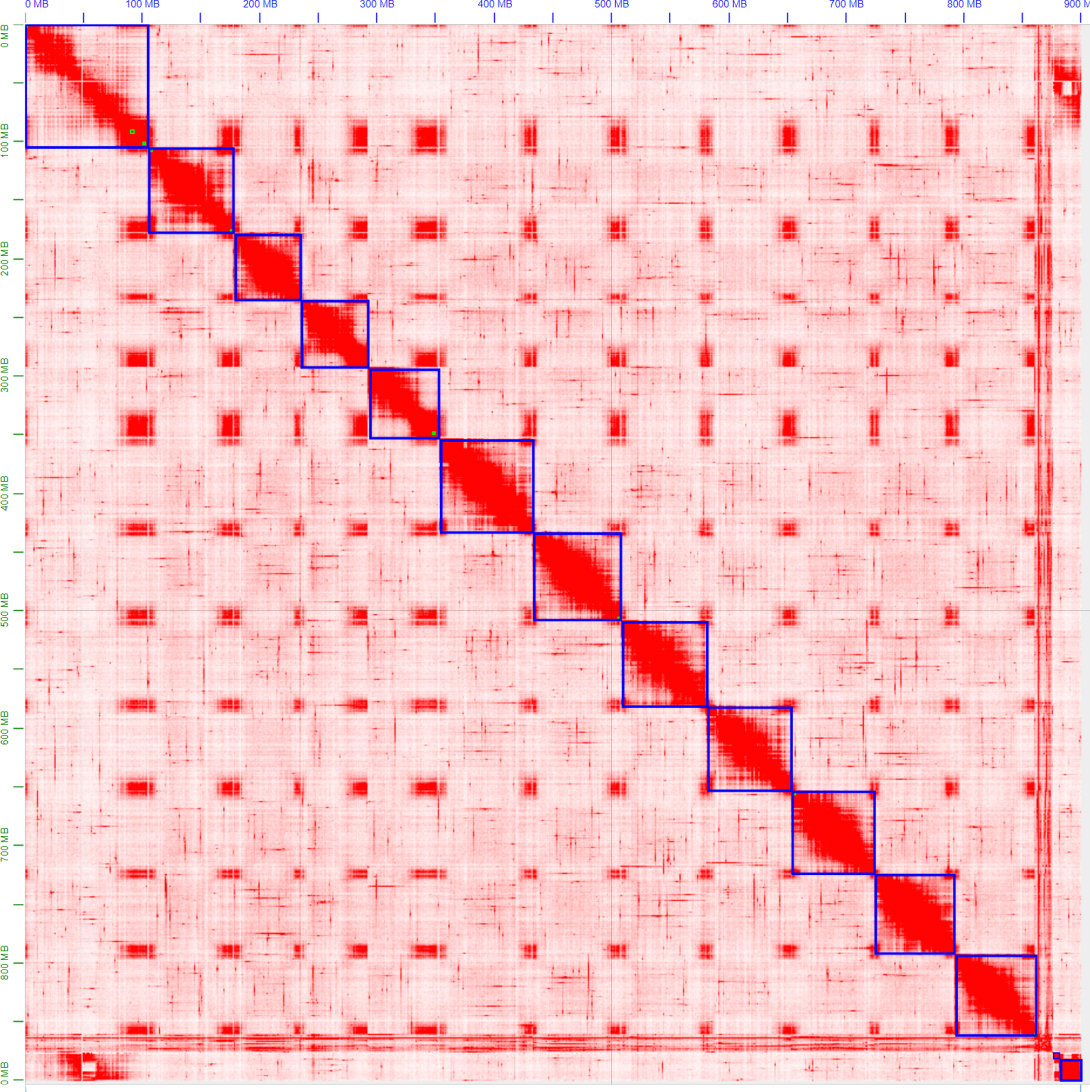
Hi-C map of the final S. chilense (LA1972) assembly, visualised in Juicebox. Hi-C contacts are represented in red, and chromosomes and scaffolds are delimited by blue squares. The squares on the bottom right represent unmapped scaffolds.

The ordering and orientation of the scaffolds with the Hi-C data resulted in a chromosome-level assembly. The assembly has an N50 of 72 Mbp and is composed of 1,911 sequences, corresponding to the 12 chromosomes from *S. chilense* and 1,899 unmapped scaffolds. Notably, 96% of the assembly was found within 12 sequence blocks. The total length of the assembly is 901 Mbp, which is close to both the estimation of 845 Mbp done via the k-mer analysis and the 914-Mbp length from a previously published assembly of the same species, but of a different accession: LA3111 (Stam et al., 2019). **Figure 5** represents the final contact map, with the pseudomolecules and unplaced scaffolds represented as blue boxes.

The assembly was estimated to be 93% complete by KAT, based on a comparison between k-mers obtained from the reads against k-mers from the assembly. This high completeness assessment was confirmed with BUSCO, which managed to identify 95% of the 3,052 orthologues from the *Solanaceae* dataset v10, as complete in the final assembly. These numbers are higher than those obtained from previously published assemblies of wild relatives of tomato, including *S. chilense*, but slightly lower than those obtained from *S. lycopersicum* and *S. pennellii*, which is expected when assembling a heterozygous, self-incompatible wild species. Comparisons of the Quast and BUSCO results with assemblies from other closely related *Solanum* species are available in **Table 3** and **Table 4** respectively. The BUSCO results obtained at each step of our pipeline are shown in **Supplementary Table 1**.

**Table 3:** Comparison of our S. chilense assembly (accession LA1972) against other tomato species.

Species	Length (Mbp)	# sequences	Largest scaffold (bp)	N50 (bp)
*S. chilense* (LA1972)	901	12 + 1,899	112,267,598	72,333,043
*S. chilense* (LA3111)	914	81,304	1,123,112	70,632
*S. pennellii*	990	12 + 4,587	109,333,515	77,991,103
*S. lycopersicum* v4.0	783	12 + 152	90,863,682	65,269,487
*S. lycopersicoides*	1,287	12 + 3,084	133,548,845	93,853,793
*S. pimpinellifolium*	826	107,698	893,636	78,865

If an assembly is at a chromosome level, the number of unplaced scaffolds is indicated by the number after the + sign.

**Table 4:** BUSCO results of our S. chilense assembly and other tomato species, based on the Solanaceae dataset v10 (C, Complete; S, Single; D, Duplicated; F, Fragmented; M, Missing BUSCOs).

Species	Complete [Single, Duplicated], Fragmented and Missing BUSCOs (*n* = 3,052)
*S. chilense* (LA1972)	C:94.9% [S:90.8%, D:4.1%], F:1.8%, M:3.3%
*S. chilense* (LA3111)	C:90.7% [S:89.5%, D:1.2%], F:4.6%, M:4.7%
*S. pennellii*	C:96.8% [S:96.1%, D:0.7%], F:1.3%, M:1.9%
*S. lycopersicum* v4.0	C:95.8% [S:94.9%, D:0.9%], F:1.7%, M:2.5%
*S. lycopersicoides*	C:93.3% [S:81.9%, D:11.4%], F:3.6%, M:3.1%
*S. pimpinellifolium*	C:93.9% [S:92.1%, D:1.8%], F:2.6%, M:3.5%

The first iteration of Pilon corrected 164,646 misassemblies, including 109,120 single-nucleotide polymorphisms (SNPs) and 55,526 InDels. The corrected assembly was then subjected to a second polishing iteration that corrected 52,653 SNPs and 22,915 InDels. Pilon detected 98.7% of correct bases in the assembly, after the two iterations were performed. The dedupe.sh script from BBMap removed 392 scaffolds, corresponding to 13 Mbp. The largest removed scaffold was 66 kbp long and 98% of the removed scaffolds were smaller than 25 kbp. GapFiller removed 237 gaps, amounting to a total of 23,964 Ns. After the GapFiller step, 12.7 Mbp of unknown bases remained in the assembly, corresponding to ~1.4% of the assembly length.

Here, we generated the first high-quality, chromosome-level assembly of *S. chilense*, which has comparable or better contiguity and completeness than other wild relatives of tomato.

### Gene prediction and annotation

RepeatMasker masked 62.63% of the assembly, which is consistent with the repeat content of similar species: 59.5% for *S. pimpinellifolium*, 64% for *S. lycopersicum* (Hosmani et al., 2019), 82% for *S. pennellii* (Bolger et al., 2014), and 70% for *S. sitiens* (Molitor et al., 2021).

STAR aligned 95.6% of the RNA-Seq reads to the masked assembly. The hints generated from the alignment allowed Augustus to predict 30,994 gene structures; this number increased to 32,972 when including alternative splicing variants. OmicsBox retrieved blast hits for 30,240 of the genes, including 22,571 annotated with GO terms. The InterProScan search identified 29,934 genes with InterProScan IDs and 18,873 genes with InterProScan GO terms.

The number of predicted genes is close to those obtained from similar species, 32,273 genes in *S. pennellii*, 34,075 genes in *S. lycopersicum*, and 31,164 genes in *S. sitiens*. Stam et al. (2019) found 41,481 genes in *S. chilense* LA3111, including 25,885 high-confidence gene models (Stam et al., 2019).

### *De novo* transcriptome assembly and annotation

Trinity generated a reference transcriptome assembly consisting of 228,844 transcripts, with an N50 of 2,685 bp and a size totalling to 360 Mbp. The BUSCO analysis identified 83.4% of the expected *Solanaceae* orthologues. After clustering the transcripts with CD-HIT-EST and filtering based on TPM counts lower than 1, the transcriptome was composed of 124,065 transcripts, with an N50 of 2,487 bp and a size totalling to 172 Mbp. The number of complete genes detected by BUSCO remained high, at 83.2%. Moreover, Bowtie2 aligned 98.59% of the RNA-Seq reads back to the assembly, further confirming the completeness of the transcriptome assembly. Of the 124,065 transcripts, 98,361 (79%) had a blast hit and 87,059 (70%) had blast top hits with an e-value ≤ 1×10^-3^ indicating these results to be a strong basis for functional annotation. GO terms were assigned to 64,028 transcripts (52%).

### Genes possessing high-impact changes and likely to be involved in abiotic stress tolerance of *Solanum chilense*

*S. chilense* contained 202 genes annotated with the GO terms related to drought, salt, or water (**Table 1**), including 43 with high-impact amino acid variants compared to *S. lycopersicum* proteins (PROVEAN score < −2.5), suggesting functional changes for the selected proteins (Choi et al., 2012). These protein variants were reverse translated back to SNPs, based on the protein MAFFT alignments and the nucleotide sequences of the genes from Augustus and ITAG4.1. Tersect, a tool to perform set operations on variant data (Kurowski and Mohareb, 2020), intersected the resulting SNPs with the 84 publicly available re-sequenced genomes of 12 tomato species (100 Tomato Genome Sequencing Consortium et al., 2014). In order to analyse fixed changes in each species, only homozygous variants were considered, which eliminated five genes from the total number.

**Figure 6:**
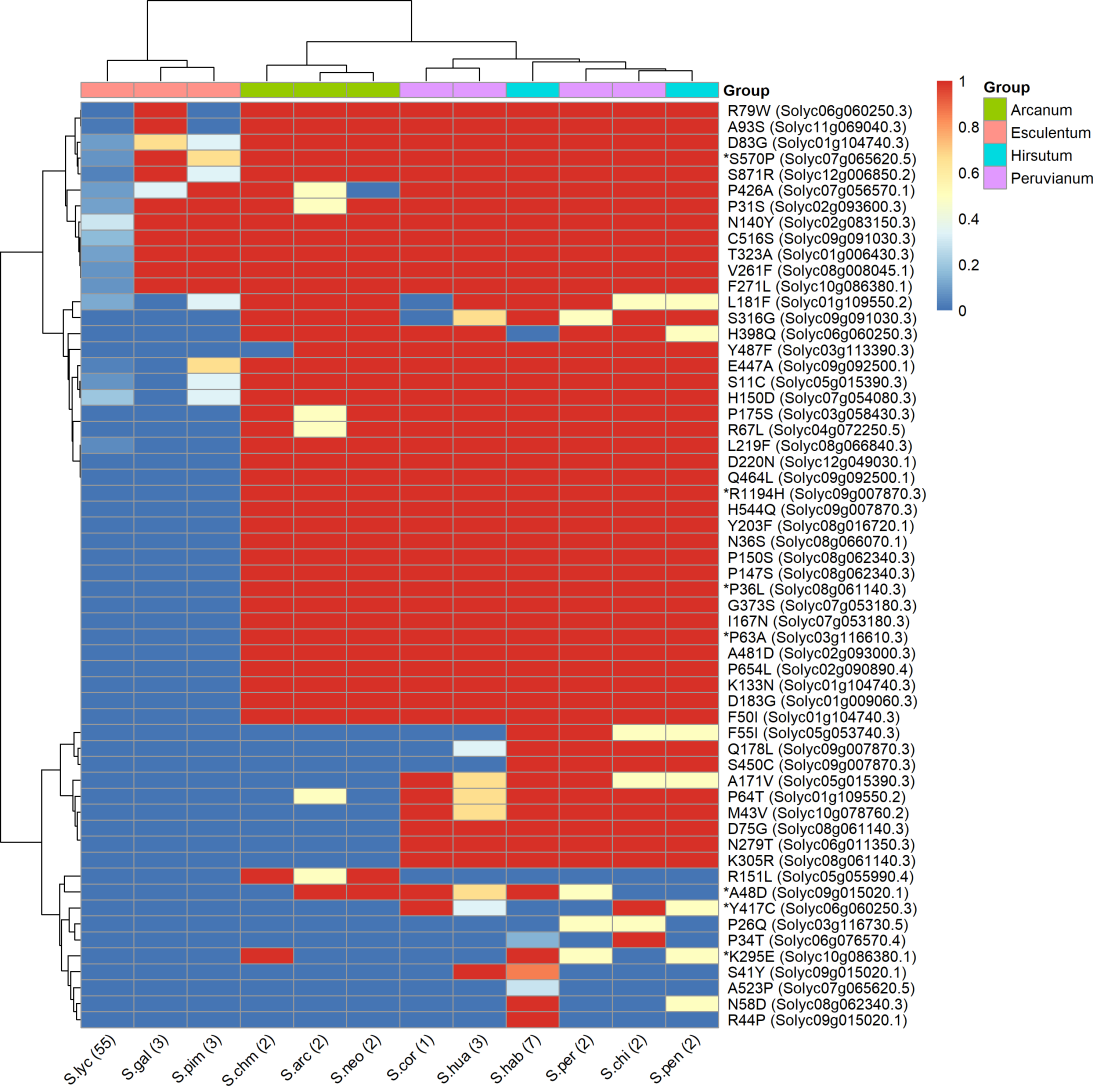
Heatmap showing the similarity of tomato and its related species based on the presence or absence of large effect variants within gene with annotation related to salinity and drought stress. Rows are the amino acid changes with a PROVEAN score < −2.5 between S. lycopersicum Heinz 1706 and S. chilense LA1972 as rows, and tomato species from the 84 tomato genomes as columns. The colour represents the proportion (between 0 and 1) of the accessions sharing a common amino acid with S. chilense. The variants, e.g., R79W, are coded as in **Table 5**. Species were allocated to each group according to taxonomy (Peralta et al., 2008). The genes identified as impactful by PROVEAN, SIFT4G, and PPVED are highlighted by an asterisk.

**Figure 6** shows the distribution of the impactful PROVEAN variants across the 12 species of tomato represented in the 84 genomes datasets. These variants are representing alleles possessing significant amino acid changes in *S. lycopersicum* compared to *S. chilense*, which highlight potential functional shift in these genes in the cultivated tomato. The clustering of the species in the heatmap matches their belonging to the taxonomic groups of genus Solanum Section Lycopersicum as defined by Pease et al. (2016), namely, the groups Esculentum, Arcanum, Peruvianum, and Hirsutum. As expected, all 57 variants, from the 38 remaining genes, are present in low percentages in the *S. lycopersicum* accessions. The impactful amino acid changes were further confirmed and reduced with the SIFT4G and PPVED algorithms; the detailed list of the resulting seven relevant genes with their corresponding variants is shown in **Table 5**, and are discussed below.

**Table 5:** List of genes with GO terms related to salt and drought, and containing impactful variants (PROVEAN < −2.5, SIFT4G < 0.05, and PPVED > 0.5, marked in bold) in *S. lycopersicum* compared to *S. chilense*.

*S. chilense* gene ID	*S. lycopersicum* ensembl ID (Annotation)	AA change	PROVEAN score	SIFT4G score	PPVED
g6365.t1	Solyc03g116610.3, Ethylene-responsive transcription factor WIN1	P63A	**−7.68**	**0.01**	**0.82**
g9938.t1	Solyc06g060250.3, Aldehyde dehydrogenase	R79W H398Q Y417C	**−3.50** **−7.31** **−8.43**	**0.00** 0.37 **0.00**	0.04 0.29 **0.51**
g18960.t1	Solyc07g065620.5, Poly(A)-specific ribonuclease PARN	S570P A523P	**−4.75** **−3.00**	**0.00** 0.41	**0.90** 0.02
g29005.t1	Solyc08g061140.3, OCP3	D75G K305R P36L	**−3.98** **−2.69** **−4.87**	0.12 0.17 **0.02**	0.07 **0.76** **0.56**
g14532.t1	Solyc09g007870.3, Ethylene-insensitive protein 2	Q178L S450C H544Q R1194H	**−5.54** **−4.32** **−2.54** **−3.93**	**0.03** **0.01** 0.34 **0.02**	0.26 0.36 0.01 **0.85**
g14972.t1	Solyc09g015020.1, class I heat shock protein 3	S41Y R44P A48D	**−2.67** **−2.79** **−4.78**	**0.02** 0.06 **0.01**	0.03 0.07 **0.68**
g27963.t1	Solyc10g086380.1, GAI-like protein 1	F271L K295E	**−5.43** **−3.70**	0.08 **0.01**	0.04 **0.79**

”AA change” gives the S. chilense LA1972 amino acid, then the protein position, and then the S. lycopersicum Heinz 1706 amino acid (e.g., P63A).

#### Solyc03g116610 (ethylene-responsive transcription factor WIN1)

The *WAX INDUCER 1* (*WIN1*) transcription factor is involved in cuticle biosynthesis in *Arabidopsis thaliana* (Aharoni et al., 2004), and its overexpression in tomato from a constitutive promoter improves drought resistance (Al-Abdallat et al., 2014) while also decreasing fruit and seed weight (Li et al., 2021). Interestingly, for the variant P63A, the proline is the last amino acid of the AP2 domain conserved across the ERF gene family (Li et al., 2021) and is present in the *S. chilense* LA1972 allele and in all accessions of the Arcanum, Eriopersicon, and Neolycopersicon groups, but it is replaced by alanine in all the Lycopersicon group accessions and hence is predicted to be deleterious to cuticle development in cultivars (**Figure 6**). Clearly, selection for the *S. chilense* allele might give improved drought resistance via reduced cuticular transpiration (Boyer, 2015), but it remains to be seen if this natural variation will also reduce fruit size as occurred with constitutive transgenic overexpression.

#### Solyc09g007870 (ethylene-insensitive protein 2)

For R1194H, the *S. chilense* LA1972 allele shares the arginine with all the Arcanum, Eriopersicon, and Neolycopersicon accessions, but histidine is present in all Lycopersicon accessions. The R1194H change is in the CEND part of the *EIN2* protein, close to the nuclear localisation signal domain (Wen et al., 2012), which directs this C-terminal part to promote gene expression changes in the nucleus (Zhang et al., 2020). *EIN2* is a large, complex protein and a component of the intracellular ethylene signalling pathway that stimulates salt tolerance in *A. thaliana* (Lei et al., 2011), and Solyc09g007870 has been proposed as a candidate gene for a QTL for rootstock-conferred drought resistance (Asins et al., 2021). Solyc09g007870 is also involved in regulating tomato fruit ripening and carotenoid accumulation (Karlova et al., 2014): a large InDel in the promoter of Solyc09g007870 reduced gene expression and was the cause of the yellow-fruited tomato 1 mutation arising in *S. pimpinellifolium* LA1585 (Gao et al., 2016). The members of the Lycopersicon group (histidine) have orange or red fruits, while the members of the Arcanum, Eriopersicon, and Neolycopersicon groups (arginine) have green fruits (Gonzali and Perata, 2021). Thus, the histidine variant may have been selected for during the evolution of the Lycopersicon group and the domestication of tomato cultivars to provide coloured fruit; it is conceivable that this may have been accompanied by a loss of resistance to drought or salinity.

#### Solyc06g060250 (aldehyde dehydrogenase; ALDH)

For the Y417C variant, the tyrosine found in *S. chilense* LA1972 is only shared with other accessions of *S. chilense* and accessions from *S. corneliomulleri, S. huaylasense*, and *S. pennellii*; all other accessions have a cysteine. The top blast hit of this gene corresponds to *aldehyde dehydrogenase family 3 member H1* of *A. thaliana*, which is highly expressed upon dehydration, in high-salinity stress and under treatment with abscisic acid (Kirch et al., 2005). The proposed function of stress-responsive ALDH3 family members is the detoxification of aldehydes that accumulate under stress as a result of lipid peroxidation; overexpression of various ALDH genes led to drought and salinity resistance (Stiti et al., 2021). Y417C is closely located to the protein C-terminus, which is responsible for its dimerisation or tetramerisation process (Shortall et al., 2021) and thus might alter ALDH enzyme function.

#### Solyc10g086380 (a GRAS transcription factor, DELLA subfamily, SlGLD1)

The *S. chilense* LA1972 allele contains two high-impact variants, which are also present in *S. chilense* LA3111 (Stam et al., 2019), but absent from the other two *S. chilense* accessions of the 84 tomato genomes dataset (CGN15530 and CGN15532). The Arabidopsis orthologues are involved in the gibberellic acid-mediated signalling and regulation of growth under environmental stresses, including drought (Wang et al., 2020) and cold (Lantzouni et al., 2020), and overexpression of *SlGLD1* in tomato gave dwarf plants (Li et al., 2015), suggesting that the gene is involved in the stress-mediated inhibition of plant growth. The *S. pennellii* LA0716 allele of *SlGLD1* was previously noted to be truncated and inactive due to InDels (Alseekh et al., 2015).

#### Solyc09g015020 (class I heat shock protein 3/SlHSP17.7B)

The overexpression of the most homologous Arabidopsis gene, *AtHSP17.8*, in lettuce resulted in dehydration and salt stress resistance phenotypes (Kim et al., 2013). However, in tomato, *SlHSP17.7B* is expressed specifically during fruit ripening, with low expression in vegetative tissues (Upadhyay et al., 2020), so there is little evidence for a role in stress tolerance in tomato.

#### Solyc07g065620 [poly(A)-specific ribonuclease PARN]

A *PARN* gene in *A. thaliana* is responsible for the regulation of appropriate status of poly(A) tract of mitochondrial mRNA (Hirayama et al., 2013) and is required for normal ABA, salicylic acid, high salinity, and osmotic stress responses (Nishimura et al., 2005). The *S. chilense* LA1972 allele of the S570P variant (serine) is present in all the accessions except some *S. lycopersicum* and *S. pimpinellifolium.* Although the S570P amino acid change appears to be outside the poly(A) polymerase (PAP) domain (Marchler-Bauer et al., 2017), the rest of the protein is highly conserved, and the amino acid change may still be significant.

#### Solyc08g061140 (Homeobox transcription factor/OVEREXPRESSOR OF CATIONIC PEROXIDASE 3; OCP3)

This gene controls an ABA-dependent drought resistance phenotype in Arabidopsis (Ramírez et al., 2009), and also mediates resistance to infection by pathogens such as *Botrytis* and *Plectosphaerella* species (Coego et al., 2005). The *S. chilense* LA1972 allele is shared with all the accessions of the Arcanum, Eriopersicon, and Neolycopersicon groups, but is absent in the Lycopersicon group. The P36L variant is close to the RNA polymerase sigma factor domain subunit (Marchler-Bauer et al., 2017) and might perturb protein function.

#### Resources to exploit genetic diversity

Mechanisms for abiotic stress resistance have evolved in wild relatives and their genetics is usually complex and quantitative; these traits can be captured for crop production, for example, in land races selected under local sub-optimal environments. However, when modern breeders focus on yield, quality, and disease resistance in near-optimal conditions, there may be erosion and bottlenecking of genetic variation for abiotic stress resistance (van de Wouw et al., 2010), and a need to actively introduce natural variation from wild relatives (Pereira et al., 2021; Kulus, 2018) with the support of genetic and genomic resources. To facilitate this, we have created a high-quality, chromosome-scale assembly and annotation of *S. chilense* (LA1972) using a range of sequencing technologies and analysis tools. We are now creating a library of introgressions derived from *S. chilense* LA1972 using the cultivar Kashi Amrit as the genetic background. This combination of genomic and genetic resources will underpin future work to understand and exploit natural genetic variation in this wild relative, and, as a first step, we have used the assembly and annotation to identify amino acid variants present in *S. chilense* LA1972 that could be targets for functional analysis and exploitation in breeding for improved drought and salt resistance. Our analysis highlighted two examples from the literature where there could be counter selection for drought or salinity resistance through pleiotropy: the “dual role” of *WIN* gene (P63A variant), which impacts drought resistance and fruit size, and the R1194H variant of *EIN2*, a gene known to influence both salinity tolerance and fruit colour.

## Data availability statement

The datasets presented in this study can be found in online repositories. The names of the repository/repositories and accession number(s) can be found below: https://www.ncbi.nlm.nih.gov/genbank/, JAPDHL000000000 https://www.ncbi.nlm.nih.gov/genbank/, PRJNA880259.

## Author contributions

CM: Data curation, Formal analysis, Software, Writing – original draft, Writing – review & editing. TK: Formal analysis, Methodology, Software, Writing – review & editing. PA: Methodology, Writing – review & editing. ZK: Validation, Writing – review & editing. DS: Formal analysis, Methodology, Writing – review & editing. SC: Formal analysis, Methodology, Writing – review & editing. JI: Formal analysis, Methodology, Writing – review & editing. PH: Funding acquisition, Investigation, Project administration, Resources, Writing – review & editing. AT: Funding acquisition, Investigation, Project administration, Resources, Supervision, Writing – original draft, Writing – review & editing. FM: Data curation, Formal analysis, Funding acquisition, Investigation, Project administration, Software, Supervision, Writing – review & editing.
