# Supplementary material for: A chromosome-level genome assembly of Solanum chilense, a tomato wild relative associated with resistance to salinity and drought
Source: Front Plant Sci. 2024 Mar 8;15:1342739. doi: 10.3389/fpls.2024.1342739 (PMC10957597; doi:10.3389/fpls.2024.1342739)
Supplement: Supplementary file 1 [file DataSheet_1.zip › Data Sheet 1 - 2024-02-21T092341.595.PDF]

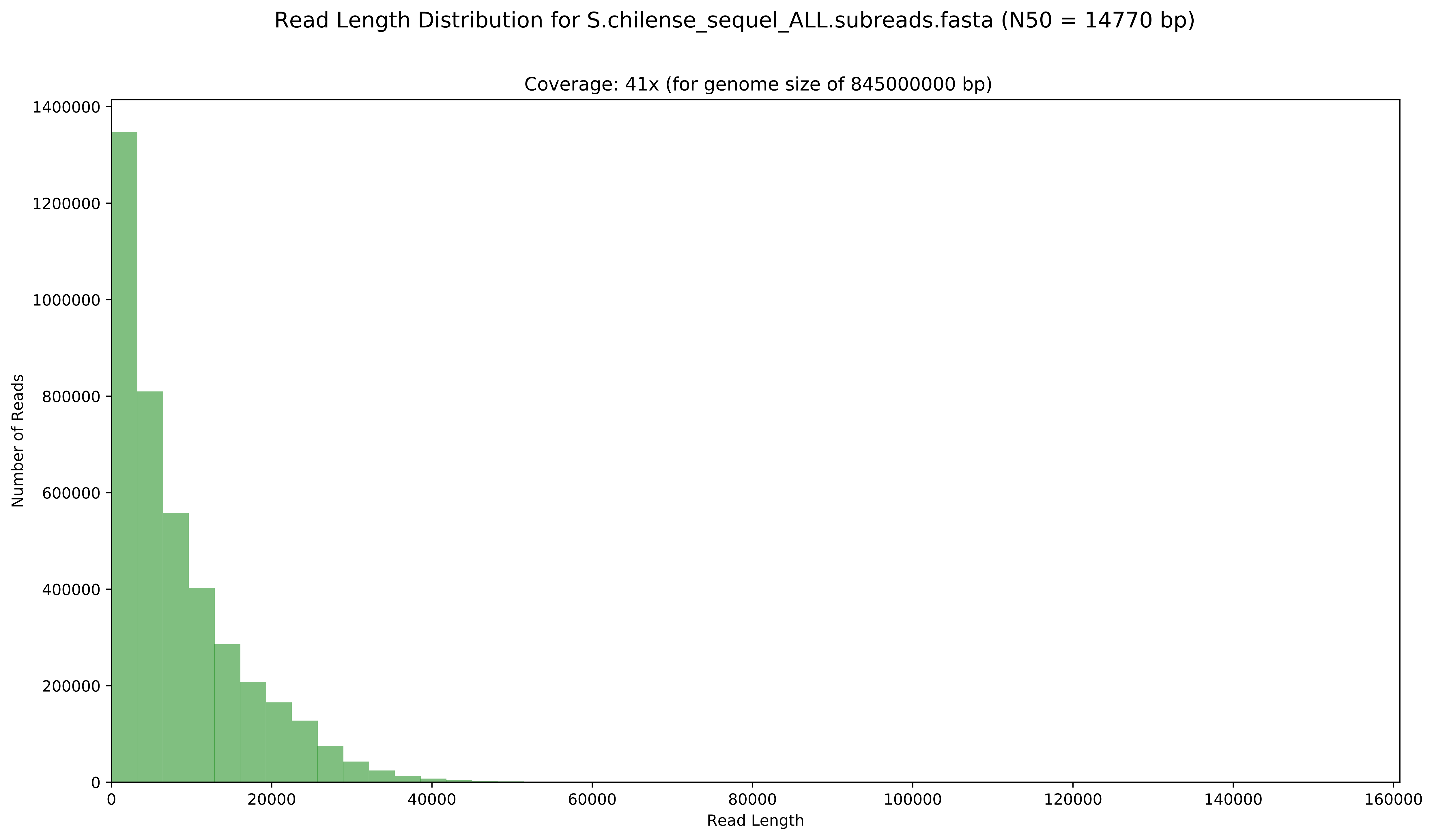
Read Length Distribution for S.chilense_sequel_ALL.subreads.fasta (N50 = 14770 bp)
Coverage: 41x (for genome size of 845000000 bp)
Number of Reads
Read Length
0
200000
400000
600000
800000
1000000
1200000
1400000
0
20000
40000
60000
80000
100000
120000
140000
160000
