# Supplementary figures and images for: A chromosome-level genome assembly of Solanum chilense, a tomato wild relative associated with resistance to salinity and drought
Source: Front Plant Sci. 2024 Mar 8;15:1342739. doi: 10.3389/fpls.2024.1342739 (PMC10957597; doi:10.3389/fpls.2024.1342739)

# Read Length Distribution for S.chilense.all.RSII.fasta (N50 = 9384 bp)

Coverage: 19x (for genome size of 845000000 bp)

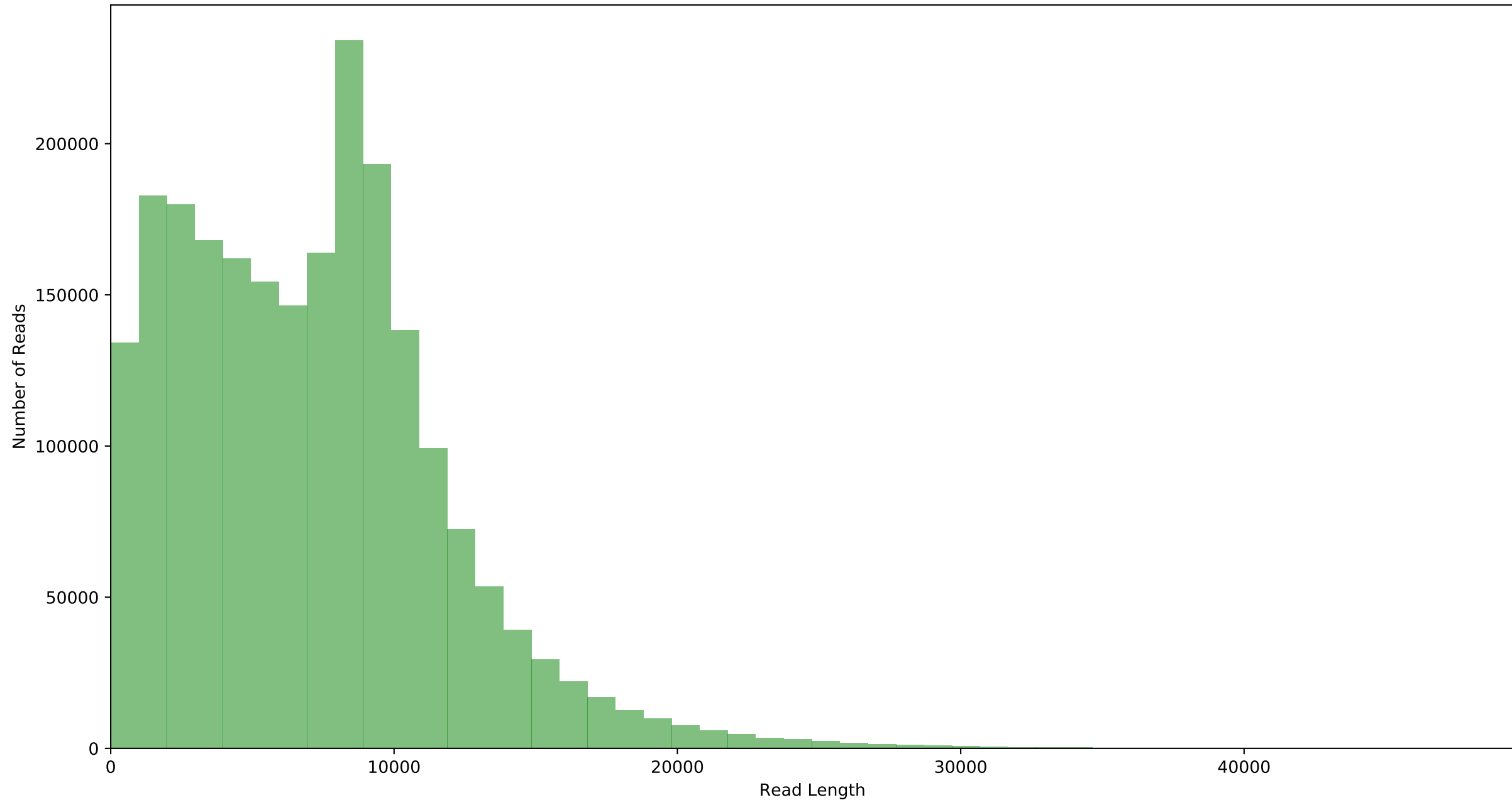

Supplement: Supplementary file 1 [file DataSheet_1.zip › Data Sheet 2 (70).PDF]
